# Totally endoscopic mitral valve replacement for mitral regurgitation post-transcatheter aortic valve implantation: a case report

**DOI:** 10.1093/ehjcr/ytaf420

**Published:** 2025-08-23

**Authors:** Yoshihiro Goto, Sho Takagi, Junji Yanagisawa, Masanori Yamamoto

**Affiliations:** Department of Cardiovascular Surgery, Toyohashi Heart Center, 21-1 Gobutori, Oyamacho, Toyohashi 441-8530, Japan; Department of Cardiovascular Surgery, Toyohashi Heart Center, 21-1 Gobutori, Oyamacho, Toyohashi 441-8530, Japan; Department of Cardiovascular Surgery, Toyohashi Heart Center, 21-1 Gobutori, Oyamacho, Toyohashi 441-8530, Japan; Department of Cardiology, Toyohashi Heart Center, 21-1 Gobutori, Oyamacho, Toyohashi 441-8530, Japan

**Keywords:** Mitral regurgitation, Transcatheter aortic valve implantation, Totally endoscopic mitral valve replacement, Case report

## Abstract

**Background:**

Mitral regurgitation (MR) may rarely worsen after transcatheter aortic valve implantation (TAVI) due to mechanical interference from the transcatheter heart valve (THV). Standard surgical approaches in these cases are often challenging due to anatomical constraints. Thus, there is a need for the development of effective alternatives to address this issue.

**Case summary:**

We present a case of a 79-year-old male on chronic haemodialysis who developed acute decompensated heart failure following implantation of a self-expanding THV. Transoesophageal echocardiography revealed anterior mitral leaflet (AML) restriction due to direct contact with the THV stent frame. A totally endoscopic mitral valve replacement (MVR), without robotic assistance, was performed via a right mini-thoracotomy. The AML was partially resected, and a 25 mm bioprosthetic valve was successfully implanted in a supra-annular position. The postoperative course was uneventful, and the patient was discharged on Day 6.

**Discussion:**

Worsening of MR after TAVI is rare but may occur due to physical interference with mitral valve leaflets. Self-expanding THVs, such as Evolut-FX, may cause leaflet restriction, especially when implanted deep or in patients with small left ventricular outflow tracts. In the present case, the stent protruded beyond the annulus, preventing leaflet motion and leading to symptomatic MR. It was demonstrated that totally endoscopic MVR is a viable minimally invasive approach for post-TAVI MR due to THV interference, even in high-risk patients.

Learning points

- Mitral regurgitation may worsen following transcatheter aortic valve implantation (TAVI) due to mechanical interaction between the transcatheter heart valve (THV) and the mitral valve.
- When mitral regurgitation is present, careful consideration is required before using self-expanding THVs, as they may increase the risk of leaflet interference.
- Totally endoscopic mitral valve replacement offers an effective and minimally invasive solution in selected high-risk patients after TAVI.

## Introduction

Transcatheter aortic valve implantation (TAVI) is an established treatment for aortic stenosis (AS), particularly in high-surgical risk patients.^1,2^ Secondary mitral regurgitation (MR) often improves after TAVI as a result of afterload reduction and subsequent left ventricular reverse remodelling.^3^ However, in rare cases, the implanted transcatheter heart valve (THV) can cause physical interference, leading to worsening of pre-existing MR.^4^ In this report, we present a case in which the stent frame of a self-expanding THV interfered with the anterior mitral leaflet (AML), exacerbating pre-existing primary MR. There is no established treatment for primary MR with morphological abnormalities induced by THV interference. We successfully performed totally endoscopic mitral valve replacement (MVR), without robotic assistance, achieving a favourable outcome.

## Summary figure

**Figure:**
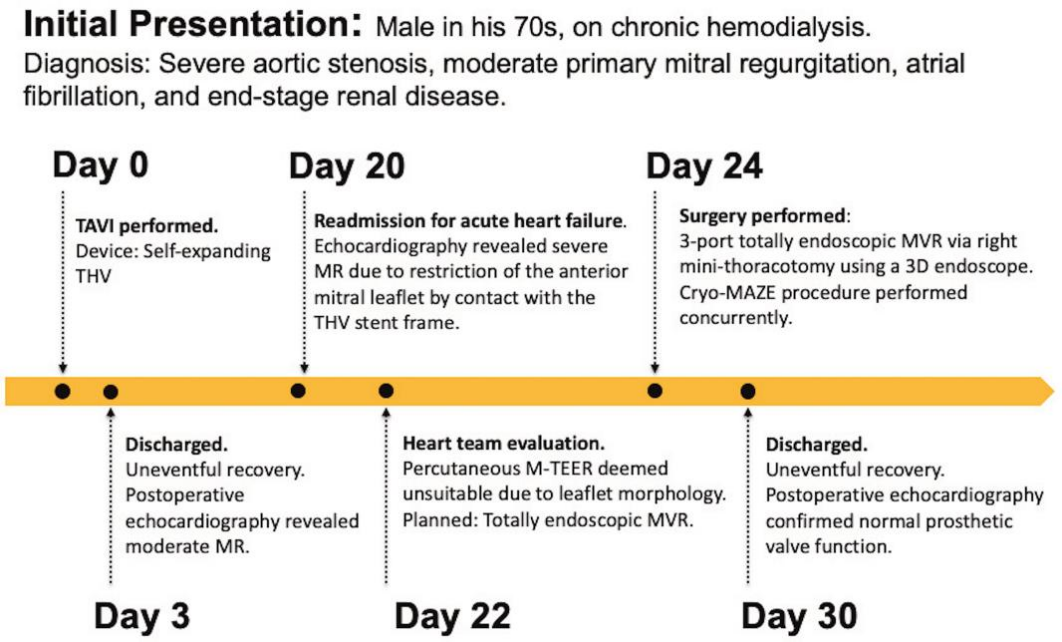


## Case report

**Figure 1:**
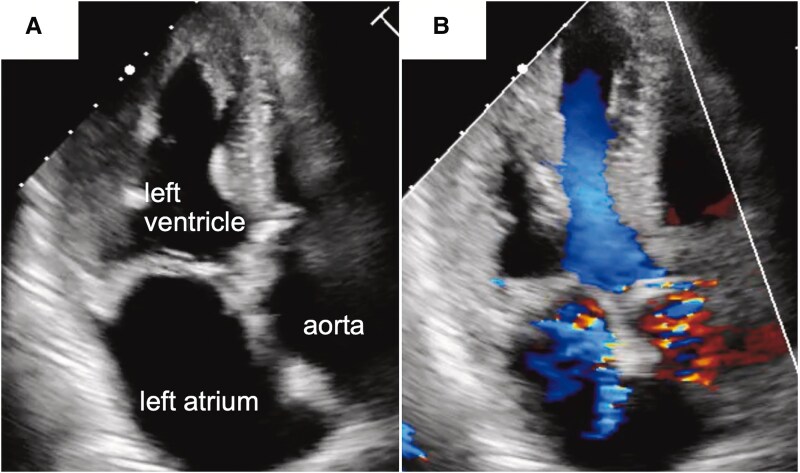
Echocardiographic views. (*A*) Pre-transcatheter aortic valve implantation transthoracic echocardiography showing severe aortic stenosis and left ventricular hypertrophy. (*B*) Doppler imaging at the same level demonstrating moderate mitral regurgitation.

The patient was a 79-year-old male on haemodialysis with severe AS, moderate primary MR owing to posterior commissure prolapse, atrial fibrillation, and end-stage renal disease (*Figure 1*).

Given the high STS risk score of 10.7, indicating elevated surgical risk associated with aortic valve replacement (AVR), a self-expanding THV (Evolut FX 26 mm, Medtronic, USA) was implanted to treat the severe AS.

**Figure 2:**
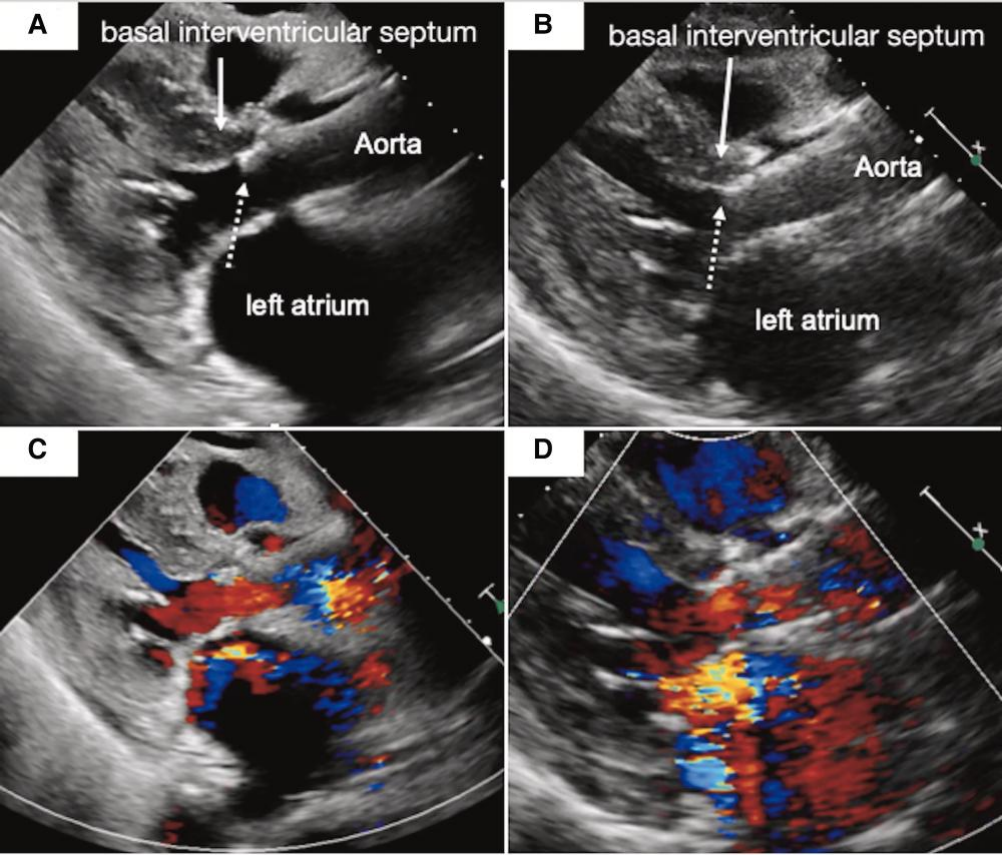
Echocardiographic views. (*A*) Post-transcatheter heart valve 2D-mode image. (*B*) Corresponding pre-transcatheter heart valve colour Doppler image. (*C*) Post-transcatheter heart valve transthoracic echocardiographic 2D-mode image obtained during heart failure exacerbation. Marked reverse remodelling of the left ventricular myocardium was observed. (*D*) Corresponding post-transcatheter heart valve colour Doppler image. The dotted arrow indicates the leftward migration of the transcatheter aortic valve implantation stent frame into the left ventricle.

**Figure 3:**
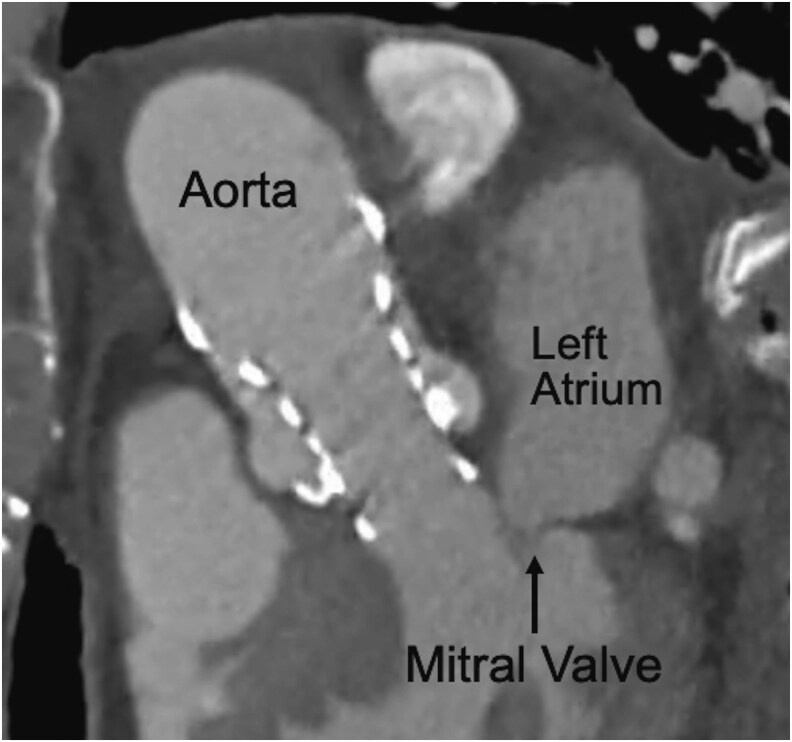
Contrast-enhanced computed tomography performed on postoperative Day 2 following transcatheter aortic valve implantation. No significant malposition or deployment issues of the TAVI valve were identified.

Post-TAVI, transthoracic echocardiography revealed no worsening of MR (*Figure 2A* and *C*). Contrast-enhanced computed tomography showed no abnormalities of the TAVI valve, including no evidence of stent migration into the left ventricle (*Figure 3*). Overall, these findings did not reveal any worsening of MR or leftward migration of the stent frame into the left ventricle. The patient showed no complications and was discharged on postoperative Day 3.

However, 20 days postoperatively, he developed heart failure due to exacerbated MR and was re-hospitalized. Transoesophageal echocardiography revealed severe MR due to restricted motion of the AML caused by THV interference (see Supplementary material online, *Video S1*).

Comparison of echocardiographic images obtained immediately after TAVI and at the time of heart failure onset demonstrated marked reverse remodelling within a short period. The left ventricular mass index decreased significantly from 158.8 to 119.3 g/m², indicating substantial regression of myocardial hypertrophy. In particular, the basal interventricular septum exhibited notable morphological changes, and the stent frame shifted towards the left ventricular cavity (*Figure 2B* and *D*). As a result, mechanical interference between the stent and the AML increased, leading to worsening of MR.

Given the complexity of the lesion extending A1–A2 segment of AML, percutaneous mitral valve transcatheter edge-to-edge repair (M-TEER) was deemed insufficient, and thus, totally endoscopic mitral valve surgery using a 3D endoscope without robotic assistance was planned.

**Figure 4:**
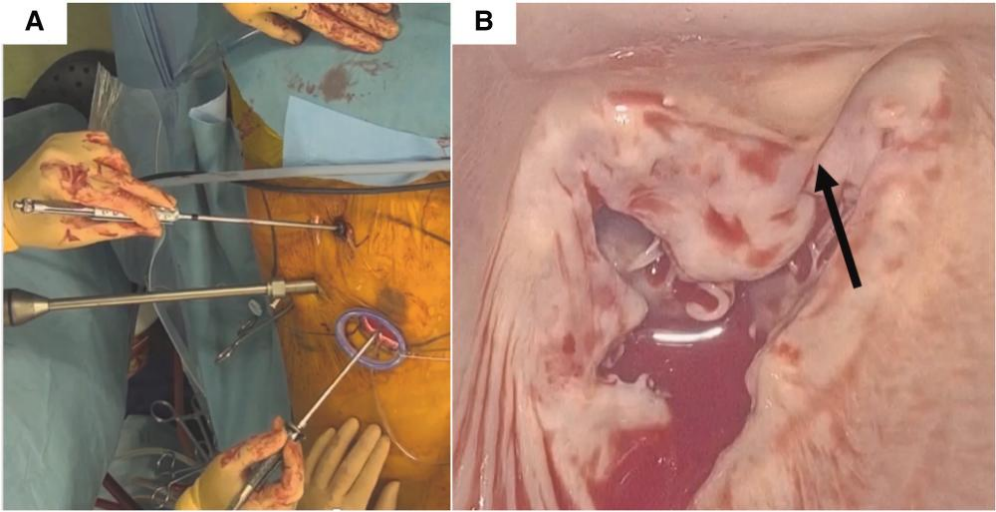
Interference of transcatheter heart valve with the anterior leaflet of mitral valve. (*A*) Intraoperative surgical set-up photograph. (*B*) Intraoperative endoscopic image. Arrow shows extension of the stent beyond the annulus.

The left arm was elevated, and a 4 cm skin incision was made at the fourth intercostal space. A 10 mm camera port was inserted at the third intercostal space, and a 5 mm instrument port was placed at the second intercostal space (*Figure 4A*). Cardiopulmonary bypass (CPB) was established via femoral arterial and venous cannulation. The distal aorta was clamped to avoid interference with the THV stent frame, and cardiac arrest was achieved with antegrade cardioplegia. A right-sided left atrial incision was performed, followed by the cryo-MAZE procedure as the initial step. Intraoperative findings showed interference of the THV stent with the AML from A1 to A2 (*Figure 4B*). Due to the complexity of the structural anomaly, mitral valve repair was deemed unfeasible, and bioprosthetic MVR was performed. The AML was resected except for a portion of A2, which remained due to interference with the THV stent. The mitral valve sutures were carefully placed in a supra-annular fashion through the mitral annulus to avoid entanglement with the THV stent, and a 25 mm Mitris bioprosthesis (Edwards Lifesciences Corporation, Irvine, CA, USA) was implanted using a non-everting mattress technique (see Supplementary material online, *Video S2*). Additionally, a portion of the AML was intentionally preserved to serve as a cushion and prevent direct interference between the mitral prosthesis and the THV stent.

The patient was uneventfully weaned from CPB, with a CPB time and an aortic cross-clamp time of 132 and 84 min, respectively. Postoperatively, he recovered without complications, and echocardiography confirmed normal prosthetic valve function (see Supplementary material online, *Video S3*). He was discharged on postoperative Day 6 with an uneventful course.

## Discussion

In general, secondary MR is known to improve following relief of AS due to afterload reduction and left ventricular reverse remodelling. However, cases of direct physical interference leading to perforation of the AML have also been documented.^5^ In the present case, pre-existing primary MR was exacerbated due to mechanical interference between the AML and the self-expanding THV stent frame.

Failure of M-TEER for post-TAVI MR cases have been reported, where the self-expanding THV was deployed too low, causing the stent frame to interfere with the mitral leaflet, thereby reducing the anterior-posterior mitral leaflet distance.^4^ In the present case, the AML was physically restricted by the stent frame, resulting in worsening MR. Although moderate MR was observed immediately after TAVI without signs of deterioration, no significant clinical concerns were noted. However, within a short period, marked reverse remodelling of the left ventricle occurred. In particular, echocardiography revealed a significant reduction in myocardial thickness at the basal septum. Given that the THV was implanted to engage the annular base, this myocardial regression likely resulted in the stent shift relatively towards the left ventricular side. Consequently, anatomical changes occurred, leading to increased interference with the AML and subsequent worsening of MR, which eventually manifested as overt heart failure.

The presence of a self-expanding THV complicates surgical exposure of the mitral valve, particularly via median sternotomy. Therefore, we opted for a totally endoscopic approach using a 3D endoscope, which provides excellent visualization and exposure of the mitral valve from a right-sided approach.^6^ Particularly, in a median sternotomy approach, observation of the mitral annulus beneath the anterior leaflet becomes difficult due to bulging of the aortic root following TAVI. In contrast, a right-sided endoscopic approach allowed for detailed inspection and safe operative manipulation. Complete resection of the anterior leaflet posed a risk of direct interference between the TAVI prosthesis and the newly implanted mitral valve prosthesis, potentially leading to valve deformation or damage. Therefore, a portion of the leaflet was intentionally retained as a cushion. Intra-annular suturing was avoided to prevent engagement with the THV stent, and instead, a supra-annular suture technique was employed. High-definition endoscopic imaging proved especially useful in confirming appropriate suture positioning to avoid stent entrapment.

Intraoperatively, we confirmed that the THV did not interfere with surgical access.

Although the stent frame protruded beyond the annulus, MVR was safely achieved by threading sutures between the stent struts in a supra-annular fashion while preserving part of the AML. Post-TAVI cardiac surgery presents challenges due to the high-risk nature of these patients and anatomical constraints caused by the implanted THV. Totally endoscopic surgery offers a minimally invasive and effective solution to mitigate these issues.

## Conclusion

We report a case of worsening primary MR following implantation of a self-expanding THV. Our findings demonstrate that a total endoscopic mitral valve surgery may provide a safe and minimally invasive approach for mitral valve intervention in complex settings.

## Supplementary Material

ytaf420_Supplementary_Data

## Data Availability

Data supporting the findings of this study are available from the corresponding author upon reasonable request.

## Lead author biography

Dr Yoshihiro Goto is a cardiac surgeon at Toyohashi Heart Center, Japan. He specializes in minimally invasive cardiac surgery and valvular heart disease, with a particular interest in mitral and aortic valve interventions. He has experience in both conventional and endoscopic approaches for valve surgery and is actively engaged in refining surgical strategies for high-risk patients. Dr Goto has contributed to several clinical cases and presentations, focusing on innovative minimally invasive surgical techniques.
